# Arnicolide D, from the herb *Centipeda minima,* Is a Therapeutic Candidate against Nasopharyngeal Carcinoma

**DOI:** 10.3390/molecules24101908

**Published:** 2019-05-17

**Authors:** Rui Liu, Brandon Dow Chan, Daniel Kam-Wah Mok, Chi-Sing Lee, William Chi-Shing Tai, Sibao Chen

**Affiliations:** 1 State Key Laboratory of Chinese Medicine and Molecular Pharmacology (Incubation), Shenzhen Research Institute, The Hong Kong Polytechnic University, Shenzhen 518057, China; rayfayliuruifei@163.com (R.L.); daniel.mok@polyu.edu.hk (D.K.-W.M.); 2 Department of Applied Biology and Chemical Technology, The Hong Kong Polytechnic University, Hung Hom 999077, Hong Kong, China; brandon.d.chan@connect.polyu.hk; 3 Department of Chemistry, The Hong Kong Baptist University, Kowloon Tong 999077, Hong Kong, China; cslee-chem@hkbu.edu.hk; 4 Institute of Medicinal Plant Development, Chinese Academy of Medical Sciences and Peking Union Medical College, Beijing 100193, China

**Keywords:** arnicolide D, NPC, anti-proliferation, cell cycle arrest, apoptosis induction

## Abstract

Nasopharyngeal carcinoma (NPC) is a high morbidity and mortality cancer with an obvious racial and geographic bias, particularly endemic to Southeast China. Our previous studies demonstrated that *Centipeda minima* extract (CME) exhibited anti-cancer effects in human NPC cell lines. Arnicolide C and arnicolide D are sesquiterpene lactones isolated from *Centipeda minima*. In this study, for the first time, we investigated their anti-NPC effects and further explored the related molecular mechanisms. The effects of both arnicolide C and arnicolide D were tested in NPC cells CNE-1, CNE-2, SUNE-1, HONE1, and C666-1. The results showed that the two compounds inhibited NPC cell viability in a concentration- and time-dependent manner. As the inhibitory effect of arnicolide D was the more pronounced of the two, our following studies focused on this compound. Arnicolide D could induce cell cycle arrest at G_2_/M, and induce cell apoptosis. The molecular mechanism of cell cycle regulation and apoptosis induction was investigated, and the results showed that arnicolide D could downregulate cyclin D3, cdc2, p-PI3K, p-AKT, p-mTOR, and p-STAT3, and upregulate cleaved PARP, cleaved caspase 9, and Bax. Regulation of cyclin B1, cdk6, and Bcl-2 expression by arnicolide D showed dynamic changes according to dose and time. Taken together, arnicolide D modulated the cell cycle, activated the caspase signaling pathway, and inhibited the PI3K/AKT/mTOR and STAT3 signaling pathways. These findings provide a solid base of evidence for arnicolide D as a lead compound for further development, and act as proof for the viability of drug development from traditional Chinese medicines.

## 1. Introduction

Nasopharyngeal carcinoma (NPC) is a type of head and neck cancer and one of the most common malignant cancers in Southeast Asia, especially in Southern China [1,2,3]. In China, NPC incidence and mortality account for 38.29% and 40.14% respectively of global cases (1.2/100,000; 0.7/100,000) [1,3]. Furthermore, the incidence and mortality of NPC are substantially higher in men than in women, and adolescents are also more likely to suffer from NPC. Additionally, the elderly, especially those over 65 years of age, exhibit relatively higher incidence and mortality rates of this cancer. The main pathogenic factors of NPC include genetic susceptibility, dietary factors, Epstein–Barr virus (EBV) infection, smoking, alcohol, and workplace exposure [4,5]. Currently, the standard primary treatment for NPC is radiotherapy, but this may cause brain radiation injury [6]. An increasing number of studies have focused on Traditional Chinese Medicines (TCMs), which have relatively minor toxic side effects [7] and significant efficacy.

Centipedae herba, the dried whole plant of *Centipeda minima* (L.) A. et Aschers. (*Ebushicao*) (Asteraceae) (CM), has been used as a TCM for thousands of years, and is commonly used for the treatment of multiple afflictions, including rhinitis, sinusitis, pain, swelling [7,8], cancer [9,10,11], allergy [12]. CM contains flavones and their glycosides, phenolic and polyphenolic acids, and sesquiterpene lactones [13,14]. Sesquiterpene lactones are built from three isoprene units, contain one or more lactone rings, and exhibit potent anti-cancer [15,16], anti-oxidant [17], and anti-inflammatory [18] activities.

**Figure 1:**
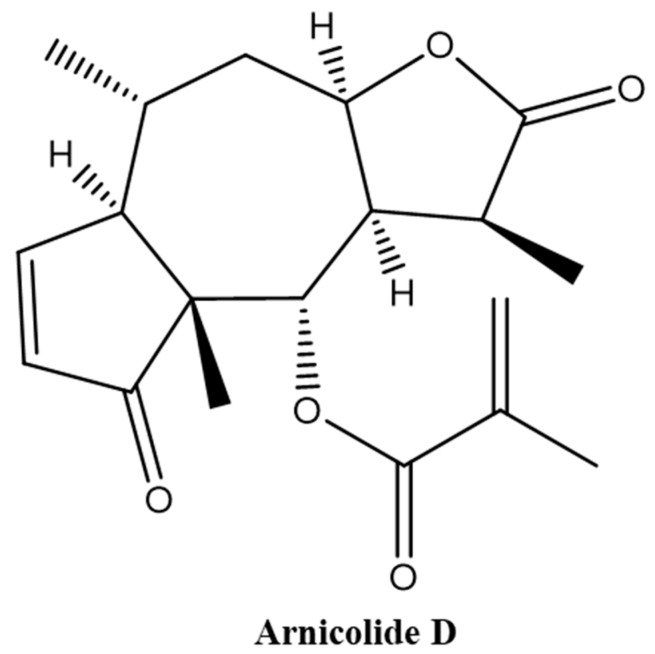
Chemical structure of Arnicolide D.

Brevilin A is a sesquiterpene lactone isolated from CM [13,14,19,20], and has been reported to exhibit multiple biological activities, including antibacterial [21] and anti-cancer [22,23] effects. The *in vitro* anti-cancer effect of brevilin A has been demonstrated in a multitude of different cancers, including human multiple myeloma, breast cancer, lung cancer, and colon cancer [15,22,23,24,25,26,27,28,29,30]. Arnicolide compounds, including arnicolide C and arnicolide D, are another type of sesquiterpene lactone found in CM [12,13,16,31,32,33]. Arnicolide D, also known as [(1*S*,3a*R*,5*R*,5a*R*,8a*R*,9*S*,9a*R*)-1,5,8a-trimethyl-2,8-dioxo-3a,4,5,5a,9,9a-hexahydro-1*H*-azuleno[6,5-*b*]furan-9-yl] 2-methylprop-2-enoate (Figure 1), has the same skeleton as brevilin A and arnicolide C. It is presumed that arnicolide D is a biologically active compound; however, only several studies investigating the biological activities of arnicolide D, especially its anti-cancer effects, exist [12,16].

Based on previous research, where CM extract demonstrated anti-cancer effects in NPC [10,11], for the first time, in this study, we investigated the anti-NPC effects of two monomer compounds, arnicolide C and arnicolide D. Our findings showed that arnicolide D could inhibit proliferation, modulate the cell cycle, and induce apoptosis in NPC cells. The anti-cancer mechanism of arnicolide D was also further investigated.

## 2. Results

### 2.1. Arnicolide D Exhibited Cytotoxic Effects on NPC Cells In Vitro

We first investigated the *in vitro* cytotoxic effects of arnicolide C and arnicolide D on the NPC cell lines CNE-1, CNE-2, SUNE-1, HONE1, and C666-1. Cells were treated with various concentrations (0–50 μM) of arnicolide C or arnicolide D for 24 h, 48 h, and 72 h. MTT assay results showed that both arnicolide C and arnicolide D exerted a cytotoxic effect on the panel of NPC cells, significantly inhibiting cell growth, in a dose- and time- dependent manner. Notably, the cytotoxic effect of arnicolide D was more potent than that of arnicolide C.

**Figure 2:**
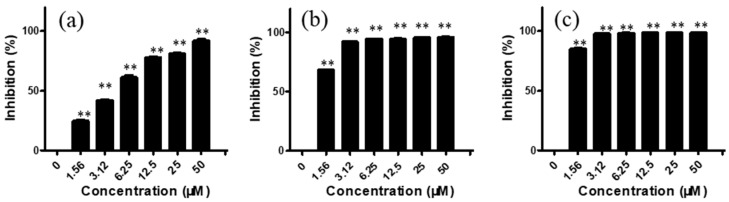
Effects of arnicolide D on proliferation of CNE-2 cells. CNE-2 cells were treated with different concentrations (0–50 μM) of arnicolide D for (**A**) 24 h, (**B**) 48 h, or (**C**) 72 h, after which MTT assay was used to evaluate the anti-proliferative effects. Cells without drug treatment were used as a control. Data are shown as means ± SD. ** *p* < 0.01, compared with control.

For CNE-2 cells, after treatment with arnicolide D (1.56, 3.12, 6.25, 12.50, 25.00, and 50 μM) for 24 h, inhibitory rates were 24.77, 42.05, 61.33, 78.03, 80.94, and 91.82%, respectively (Figure 2). Similar to trends after 24 h treatment, inhibitory rates were also increased with increasing concentrations of arnicolide D after 48 h and 72 h treatment. The inhibitory activity of arnicolide D on CNE-2 cells also increased along with treatment time. Arnicolide D at 1.56 µM inhibited cell growth by 24.77% at 24 h, 68.58% at 48 h, and 85.20% at 72 h. The calculated IC_50_ values of arnicolide D in CNE-2 cells with treatment times of 24 h, 48 h, and 72 h were 4.26, 0.99, and 0.83 µM, respectively (Table S2). Likewise, the inhibitory effect of arnicolide D in other NPC cells increased with dose and time. Arnicolide C also exhibited inhibitory effects on NPC proliferation, but at a smaller magnitude than that of arnicolide D. The IC_50_ values of arnicolide C in CNE-2 cells were 12.3 µM at 24 h, 4.64 µM at 48 h, and 3.84 µM at 72 h (Table S1).

### 2.2. Cell Morphology

**Figure 3:**
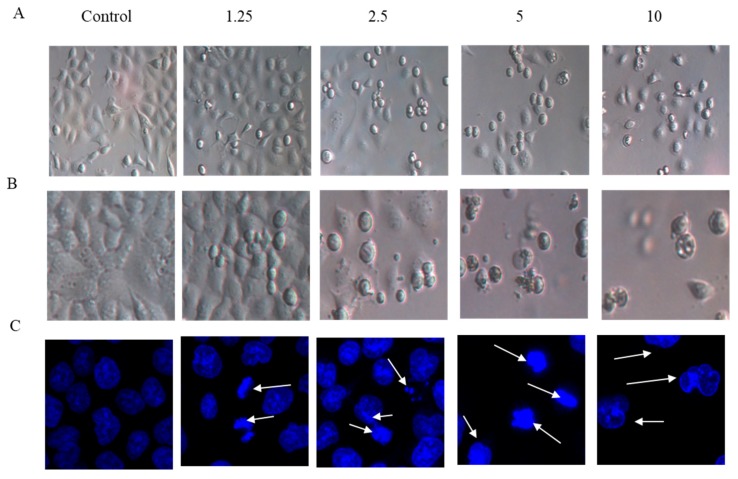
Morphological changes of CNE-2 induced by arnicolide D. CNE-2 cells were treated with different concentrations of arnicolide D (2.5–10 μM) for (**A**) 24 h or (**B**) 48 h and cell morphology was observed under an optical microscope (magnification, 100×). (**C**) After 48 h treatment, cells were stained with DAPI and their nuclear morphologies were observed using confocal microscopy (magnification, 400×).

The effects of arnicolide D on the morphology of NPC cells were observed under light microscopy (Figure 3A,B), or observed after DAPI staining with confocal microscopy (Figure 3C). CNE-2 cells from control groups (24 h and 48 h) showed normal cell architecture with clear cytoskeletons, while cells from arnicolide D treatment groups exhibited typical morphological changes associated with apoptosis, including cell shrinkage, increased chromatin condensation, visible formation of apoptotic bodies, and nuclear degradation (Figure 3, white arrows). Morphological changes were increasingly pronounced with increased doses of arnicolide D. Here, results indicated that arnicolide D induced apoptosis in a dose-dependent manner.

### 2.3. Arnicolide D Modulated Cell Cycle Distribution in NPC Cells

To determine the effect of arnicolide D on the cell cycle of NPC cells, CNE-2 cells were treated with arnicolide D at concentrations of 1.25, 2.5, 5, 7.5, and 10 µM for 24 h or 48 h, and analyzed by flow cytometry.

**Figure 4:**
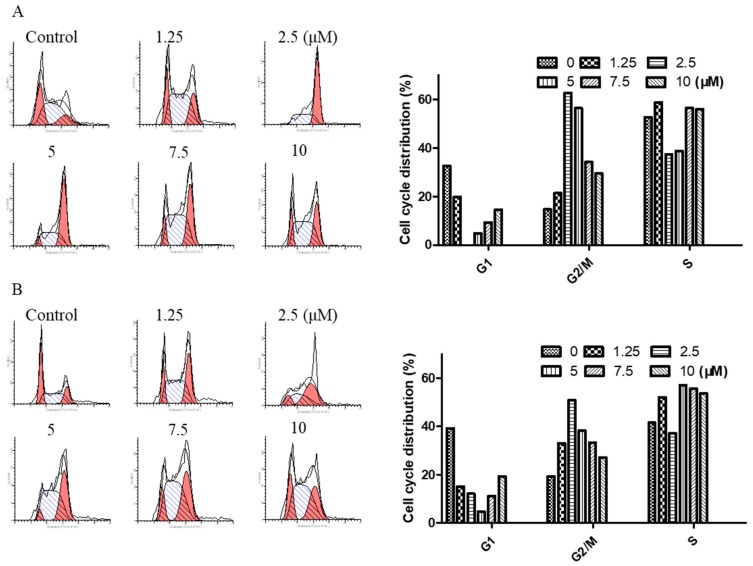
Arnicolide D induced cell cycle arrest at the G_2_/M phase. CNE-2 cells were treated with arnicolide D at 1.25–10 μM for 24 h or 48 h and then assessed via flow cytometry. Representative DNA fluorescence histograms of PI-stained cells showing the cell cycle distribution for (**A**) 24 h or (**B**) 48 h. (**C**, **D**) Bar graphs show the proportion of CNE-2 cells in different phases. Values are shown as means ± SD and are representative of three independent experiments.

Results showed that cells were significantly arrested at G_2_/M after 24 h and 48 h treatment with arnicolide D (Figure 4). G_2_/M cells were dramatically increased in a dose-dependent manner, and the fraction of G_2_/M phase cells reached its maximum (approximately 62.63% at 24 h and 50.83% at 48 h) at a dose of 2.5 µM arnicolide D. This increase was correspondingly accompanied by a significant decrease in G_1_ phase cells. Taken together, these results exhibited the cell cycle modulatory activity of arnicolide D in NPC cells, which may relate to its anti-proliferative and apoptosis-inducing effects.

### 2.4. Flow Cytometric Analysis of Apoptosis

**Figure 5:**
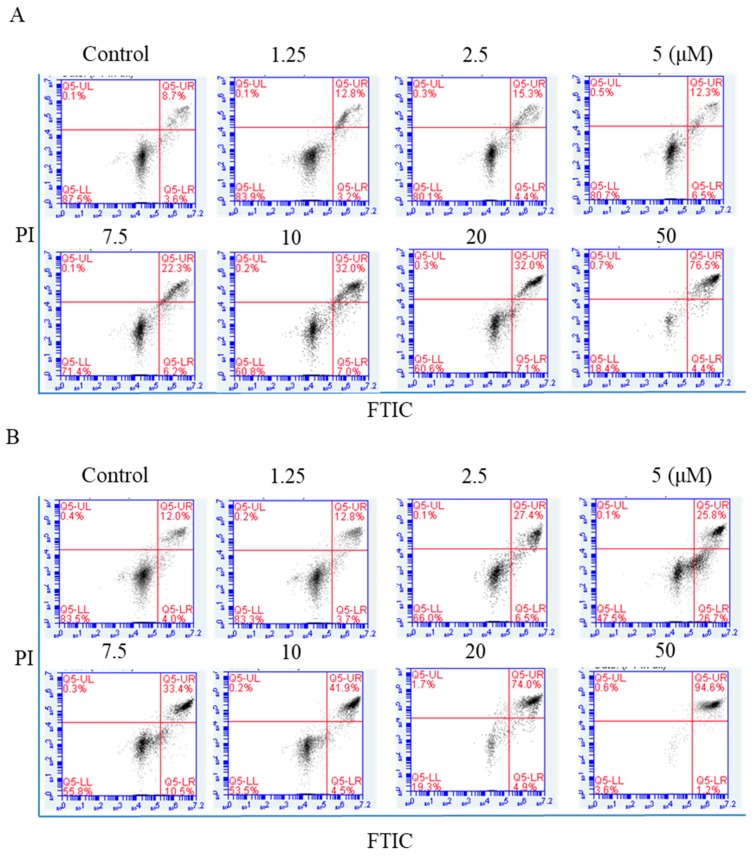
Flow cytometric analysis of arnicolide-D-induced apoptosis. CNE-2 cells were treated with arnicolide D at 1.25–10 μM for 24 h or 48 h, stained with Annexin-V/PI, and analyzed by flow cytometry. Representative images showing apoptotic cell populations after (**A**) 24 h or (**B**) 48 h treatment. Quantification of apoptotic cell populations, including early apoptotic (bottom right quadrant) and late apoptotic (top right quadrant) cells are shown.

NPC cells were exposed to arnicolide D (1.25, 2.5, 5,7.5, 10, 20, or 50 µM) for 24 h, and annexin V-FITC/Propidium iodide (PI) double staining was carried out to detect apoptosis by flow cytometry. Apoptotic cell percentages ranged from 11.3% to 65.8% after arnicolide D treatment (Figure 5A). After 48 h treatment, the percentage of apoptotic cells ranged from 11.03% to 59.87% (Figure 5B). NPC cells treated with arnicolide D exhibited significant increases in early and late apoptotic cell populations, in a dose- and time-dependent manner when compared to controls without treatment. These results clearly suggested that arnicolide D induced apoptosis in NPC cells.

### 2.5. Western Blot Analysis of Cell-Cycle-Related Proteins

**Figure 6:**
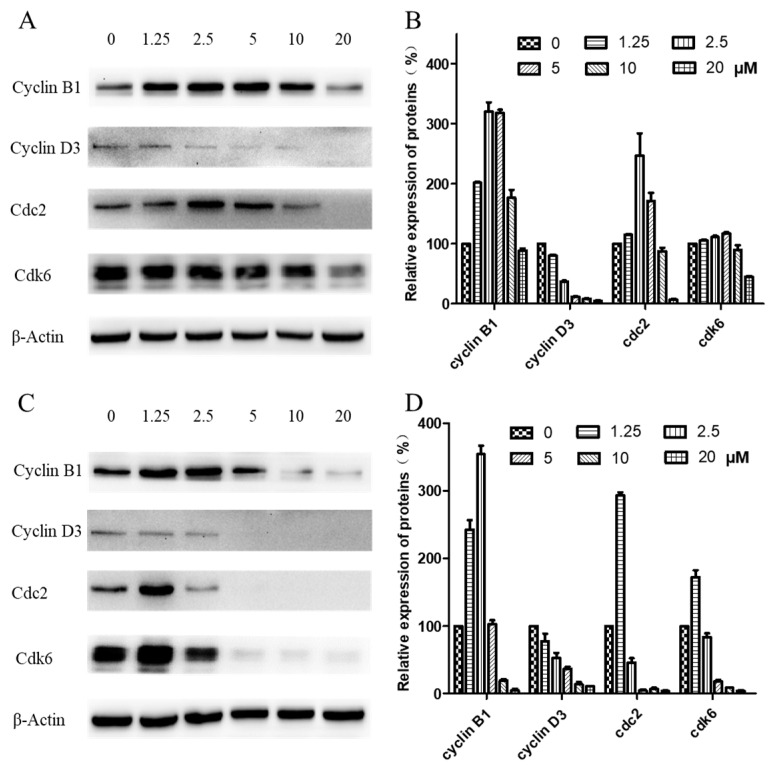
Effects of arnicolide D on the expression of key cell cycle proteins. CNE-2 cells were treated with arnicolide D at concentrations of 1.25–20 μM for 24 h or 48 h, and cell lysates were harvested and subjected to Western blot analysis using antibodies against cyclin B1, cyclin D3, cdc2, and cdk6. β-actin was used as an internal control. Western blot results are shown for (**A**) 24 h or (**C**) 48 h treatments. Bar graphs (**B**,**D**) showing the quantified relative expression of the proteins. Data are expressed as means ± SD.

Western blotting was carried out to examine expression levels of cyclin B1, cyclin D3, cdk1/cdc2, and cdk6, key regulators of the G_2_/M checkpoint. As shown in Figure 6, when CNE-2 cells were treated with arnicolide D at concentrations from 0 to 20 µM for 24 h (Figure 6A,B) or 48 h (Figure 6C,D), expression levels of cyclin D3 were decreased in comparison with control cells. Additionally, expression levels of cyclin B1, cdk1/cdc2, and cdk6 were first dose-dependently increased, and then significantly decreased in arnicolide-D-treated cells.

### 2.6. Expression of Proteins Related to Apoptosis

**Figure 7:**
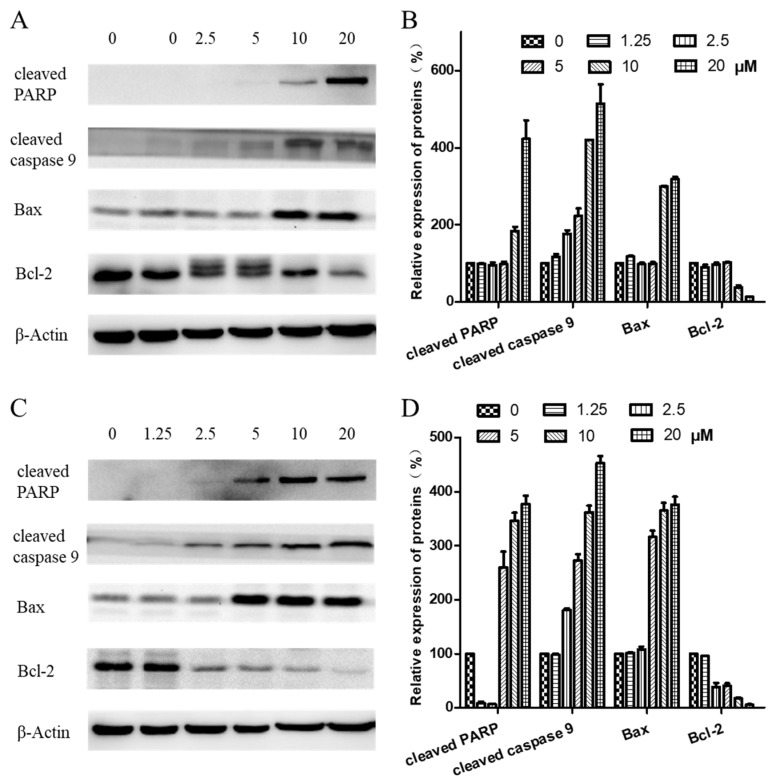
Effects of arnicolide D on the expression of apoptotic proteins. CNE-2 cells were treated with arnicolide D at concentrations of 1.25–20 μM for 24 h or 48 h, and cell lysates were harvested and subjected to a Western blot analysis using antibodies against cleaved PARP, cleaved caspase 9, Bax, and Bcl-2. β-actin was used as an internal control. Western blot results are shown for (**A**) 24 h or (**C**) 48 h treatments. Bar graphs (**B** and **D**) showing the quantified relative expression of the proteins. Data are expressed as means ± SD.

To further investigate the mechanism of arnicolide-D-induced apoptosis in NPC cells, expression of apoptosis-related proteins, including cleaved poly (ADP-ribose) polymerase (cleaved PARP) (p85), cleaved caspase 9, Bax, and Bcl-2, were evaluated by Western blot. After treatment with arnicolide D (1.25–20 µM) for 24 h (Figure 7A,B) or 48 h (Figure 7C,D), levels of cleaved PARP (p85), cleaved caspase 9, and Bax were dramatically increased. Interestingly, expression of the anti-apoptotic protein Bcl-2 was downregulated in a dose-dependent manner at 48 h.

### 2.7. Regulation of the PI3K/AKT and STAT3 Signaling Pathway

**Figure 8:**
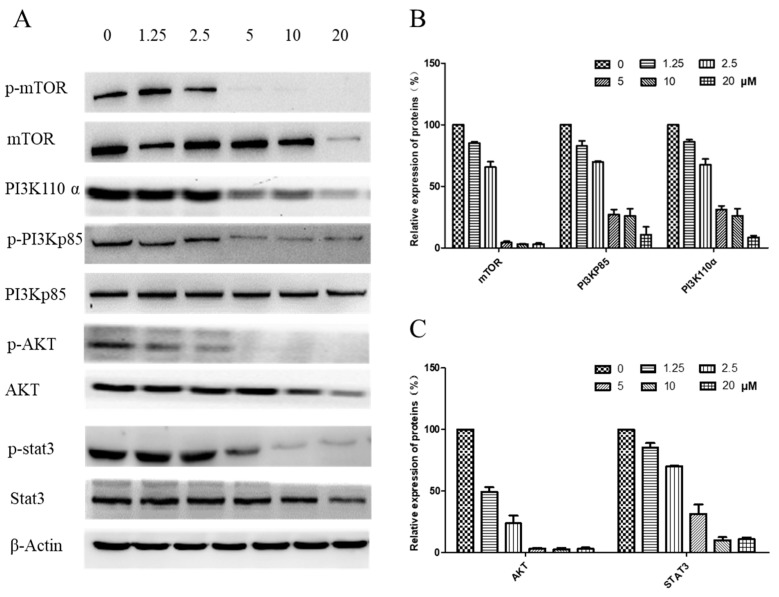
Effects of arnicolide D on the PI3K/AKT pathway (48 h). CNE-2 cells were treated with arnicolide D at concentrations of 1.25–20 μM for 48 h, and cell lysates were harvested and subjected to Western blot analysis using antibodies against mTOR and p- mTOR (Ser^2481^), PI3K p110 α, PI3K p85 and p- PI3K p85 (Tyr^458^), AKT and p- AKT (Ser^473^), and STAT3 and p-STAT3 (Tyr^705^). β-actin was used as an internal control. (**A**) Western blot results after 48 h treatment. (**B**,**C**) Bar graphs showing quantified relative expression of the proteins. Data are expressed as means ± SD.

The underlying pathway involvement in arnicolide-D-induced apoptosis was further explored, and we assessed whether arnicolide D treatment could downregulate the expression of key proteins of the PI3K/Akt/mTOR pathway. After treatment with arnicolide D at various concentrations for 24 h (Figure S3) and 48 h (Figure 8), the levels of PI3K p110α, p-PI3K p85, p-Akt, and p-mTOR were notably and dose-dependently decreased when compared to control. Investigation of the effects of arnicolide D on STAT3 signaling showed that the compound could inhibit the protein expression of p-STAT3, the key member of the pathway.

## 3. Discussion

NPC displays a clear racial and geographic bias, and is much more common in certain parts of North Africa and Asia, particularly Southeast China [34,35]. The high incidence of NPC in Southern China and Hong Kong has led to its alternate designations, ‘Guangdong Cancer’ or ‘Guangdong Tumor’ [1,3,34]. NPC has been associated with high mortality and morbidity, burdening patients not only with heavy economic pressure, but also great psychological pressures. 

TCMs comprise a host of naturally occurring herbal medicines, and represent a major facet of Chinese culture and civilization. As TCMs tend to exhibit relatively few-to-no side effects, their use in the prevention and treatment of NPC has been increasingly reported.

*Centipeda minima* (CM) is a TCM usually used for treatment of rhinitis by herbalist doctors in China. Most modern research shows that CM possesses anti-bacterial, anti-inflammatory, and anti-cancer effects [7,9]. Our previous studies have shown that *C. minima* ethanol extracts (CME) could inhibit the proliferation of CNE-1 cells and activation of the PI3K/AKT/mTOR signaling pathway [11].

Brevilin A is a sesquiterpene lactone isolated from CM, and possesses a variety of pharmacological activities [13,14,36]. Arnicolide C and arnicolide D are also sesquiterpene lactones isolated from CM [1,12,13,32,33], however, relatively few studies have been conducted to investigate their biological activity. In this study, we showed that arnicolide D could inhibit the proliferation and viability of NPC cells in a time- and dose-dependent manner. Treatment of NPC cells with arnicolide D led to changes in cell morphology, including cell shrinkage and development of vacuoles. DAPI staining identified sub-lobular cell nuclei, with fragmented or fringed shapes. Altogether, these morphological changes suggest that arnicolide D could induce apoptosis.

Cell cycle control is a complex and elaborate process, and defects in its modulation may result in abnormal metabolism and proliferation. It has been reported that arnicolide C and D could modulate the cell cycle in HT-29 human colon cancer cells [16]. Via flow cytometry analysis, we demonstrated that arnicolide D could notably induce G_2_/M phase arrest in NPC cells in a dose- and time-dependent manner, which was in line with the activity of the related compound, brevilin A [30]. As G_2_/M is critical to cell cycle progression, the molecular mechanism of cell cycle regulation caused by arnicolide D was further investigated. Cyclin B1 plays an important role in G_2_/M transition as well as M phase progression. The expression of cyclin B1 protein is increased in the G_2_ phase and rapidly decreased during anaphase, which may be associated with inhibition of mitosis [37,38]. Arnicolide D upregulated the expression of cyclin B1 at concentrations from 1.25 to 5 µM, while decreasing its expression at concentrations from 5 to 20 µM. This increase, followed by decrease in cyclin B1 may be due to a switch from a cell cycle-regulatory dose to an apoptosis-inducing dose with increasing concentrations of arnicolide D. The above phenomenon is consistent with the results of our cell cycle analysis, where G_2_/M phase cells first increase, and then decrease with increasing concentrations of arnicolide D. Previous studies have shown that the expression of cyclin B1 was altered after arnicolide D treatment in a dose- and time- dependent manner [25,26]. Additionally, increased expression of cyclin B1 has been shown to result in G_2_/M arrest [25]. 

Overexpression of cdc2 results in over-proliferation, which can lead to tumor formation. Cdc2 interacts with cyclin B1 and forms maturation promoting factor (MPF), a key protein in mitotic regulation, at the late G_2_ stage. In the present study, the protein expression of cdc2 was reduced after arnicolide D treatment, implying that cdc2 participated in arnicolide-D-induced G_2_/M arrest. Moreover, arnicolide D decreased the expression of cyclin D3 and regulated the expression of cdk6. This is in line with previous articles, which also showed that sesquiterpene lactones could inhibit the expression of cyclin D3 [30].

Apoptotic induction in cancer cells is a crucial therapeutic strategy for cancer treatment. After treatment of arnicolide D, NPC cells demonstrated hallmark morphological changes associated with apoptosis. This induction of apoptosis was confirmed by flow cytometry using an annexin V-FITC/PI double-staining approach. After treatment of arnicolide D, the proportion of early and late apoptotic NPC cells was increased when compared to control, suggesting that arnicolide D was able to induce NPC cell apoptosis. Our findings are in line with previous studies, where the related sesquiterpene lactone, brevilin A, was shown to induce apoptosis in cancer cells [15,27,28].

Expression of apoptosis-related proteins was then evaluated by Western blotting to investigate the mechanisms of arnicolide-D-induced apoptosis. Apoptosis ultimately leads to a change in the expression of cleaved caspases, and in previous studies, the related compound brevilin A had been shown to modulate caspase regulation in human multiple myeloma cells [23]. After treatment with arnicolide D, the expression of cleaved caspase 9 in CNE-2 cells was increased. Expression of cleaved PARP (p85) was also increased, altogether suggesting the caspase apoptotic pathway as a mechanism for arnicolide-D-induced cell apoptosis. This is consistent with previous studies that showed increased cleaved caspase 9 and cleaved PARP in apoptotic cells [27,39].

The B cell lymphoma 2 (Bcl-2) family includes death antagonists, such as Bcl-2 and Bcl-xl, and death agonists, such as Bax and Bad, which play an important role in the regulation of mitochondrial-mediated apoptosis. Increased expression of Bcl-2 occurs in a variety of human cancer cells and tissues, where it can suppress a number of apoptotic death programs. Brevilin A was reported to induce mitochondrial apoptosis in U87 glioblastoma cells and cause increased expression of cleaved caspase 9 and cleaved PARP [27]. Arnicolide C and D have also been reported to induce cancer cell apoptosis in the HT-29 human colon cancer cell line [16]. In this study, arnicolide D downregulated the expression of Bcl-2, and upregulated the expression of Bax, resulting in CNE-2 cell apoptosis.

Proliferation and apoptosis of tumors are regulated by multiple signaling pathways, such as the PI3K/AKT/mTOR, MAPK/ERK, and STAT3 pathways [11,24,40,41,42]. The activation or inhibition of these signaling pathways are not isolated but interrelated and interact with each other, each a node in a network of relations. The PI3K/AKT/mTOR pathway is essential for the regulation of cell survival and apoptosis [43]. Previous reports have shown that brevilin A could inhibit the PI3K/AKT/mTOR pathway to induce apoptosis and autophagy in multiple cancer types [10,11,12,13,14,15,16,17,18,19,20,21,22,23,24,25,26,27,28]. In this study, we showed that arnicolide D could suppress PI3K/AKT/mTOR activity in CNE-2 cells, which may be a potential molecular mechanism associated with its inhibition of NPC cell proliferation, and induction of apoptosis.

STAT3 signaling was also investigated in this study. The level of STAT3 in NPC tissues has been shown to be higher than that in normal tissues, and is negatively correlated with survival rates [44]. The related compound, brevilin A, has been reported to decrease the expression of p-STAT3 in many types of cancers, including lung, breast, and liver [22,24]. In this study, arnicolide D inhibited the expression of p-STAT3 in CNE-2 cells. This inhibitory effect may contribute to the induction of apoptosis and suppression of migration in cancer cells [24]. Inhibition of STAT3 activation can also lead to the inhibition of AKT activation [22], which was also reflected our findings, indicating the potential involvement of multiple signaling pathways in the anti-cancer activity of arnicolide D.

## 4. Conclusions

In the present study we showed that arnicolide D inhibited proliferation and induced apoptosis in NPC cells. The mechanisms of arnicolide D may involve promotion of cell cycle arrest at the G2/M phase, activation of the PI3K/AKT/mTOR signaling pathway, and induction of the mitochondrial apoptosis pathway.

The anti-proliferative and apoptosis-inducing anti-cancer activity of arnicolide D in NPC likely depends on interactions between members of a network of signaling pathways. In this paper, our findings have demonstrated the anti-NPC effect of arnicolide D, and lay down groundwork for further clinical and pathological research in NPC.

## 5. Materials and Methods

### 5.1. Drugs and Reagents

Arnicolide C and arnicolide D were purchased from Jiangsu Yongjian Pharmaceutical Co., Ltd. (Jiangsu, China). Cisplatin was obtained from the National Institutes for Food and Drug Control (Beijing, China). Dulbecco’s Modified Eagle’s Medium (DMEM), Dulbecco’s phosphate-buffered saline (DPBS), fetal bovine serum (FBS), penicillin streptomycin, and 0.25% trypsin-EDTA were purchased from Gibco (Gibco Life Technologies, Grand Island, NY, USA). Thiazolyl blue tetrazolium bromide (MTT), dimethyl sulfoxide (DMSO), and bovine serum albumin were purchased from Sigma (St. Louis, MO, USA). Annexin V-FITC cell apoptosis detection kits and Cell cycle detection kits were obtained from Beyotime Institute of Biotechnology (Shanghai, China). Polyvinylidene difluoride transfer membranes were sourced from Merck Millipore Ltd. (Bedford, MA, USA). The Pierce Bicinchoninic acid (BCA) protein assay kit was obtained from Thermo Scientific Inc. (Rockford, IL, USA).

Skim milk, TBS buffer, and Western blotting substrate were obtained from Solarbio Life Sciences and Sangon Biotech. Primary antibodies, including PI3K, AKT, mTOR, Bcl-1, Bax, cyclin B1, cyclin D3, and CDK6, and secondary antibodies were purchased from Cell Signaling Technology Inc. (Beverly, MA, USA). β-actin antibody was purchased from Zsbio Commerce Store (Beijing, China). A Tanon High-sig ECL Western Blotting Substrate kit was purchased from Tanon Science & Technology Co., Ltd. (Shanghai, China).

### 5.2. Cell Culture and Drug Treatments

Human nasopharyngeal carcinoma cells CNE-1, CNE-2, SUNE-1, HONE1, and C666-1 were supplied by the Hong Kong NPC AoE Cell Line Repository.

CNE-1, CNE-2, SUNE-1, and HONE1 cells were grown in Dulbecco’s Modified Eagle’s Medium (DMEM) with 10% heat-inactivated fetal bovine serum (FBS) and 1% Penicillin/Streptomycin. C666-1 cells were grown in Roswell Park Memorial Institute medium (RPMI) 1640 with 10% FBS and 1% Penicillin/Streptomycin. All cells were maintained in an incubator with a humidified atmosphere and 5% CO_2_ at 37 °C.

### 5.3. Cell Viability Assay

The cytotoxic effects of arnicolide C and arnicolide D on NPC cells were assessed using the MTT cell viability assay [11,40]. Cell viability was measured by the reduction of 3-(4,5-dimethylthiazol-2-yl)-2,5-diphenyltetrazolium bromide (MTT) to formazan. Cells were seeded overnight (2000 cells/well for CNE-1, CNE-2, and SUNE-1; 4000 cells/well for HONE1; and 15,000 cells/well for C666-1) in 96-well plates. After overnight incubation, cells were treated with various concentrations of arnicolide C or arnicolide D for 24 h, 48 h, or 72 h (0–50 µM). MTT reagent was then added to the medium in each well and incubated for 4 h at 37 °C. Reduced formazan crystals were solubilized in DMSO and optical density values were measured at 570 nm on a microplate reader. Proliferation inhibition rates of arnicolide C and arnicolide D were calculated using the following equation:

$$Inhibition\left(\%\right)=[1-(OD_{drug}-OD_{blank})/\;(OD_{control}-OD_{blank})]\times 100.$$

Six parallel wells were tested for each condition. An average value was obtained from three replicate experiments. Half-maximal inhibitory concentration (IC_50_) values were calculated using GraphPad Prism software v5.0 (GraphPad Software, San Diego, CA, USA).

### 5.4. Cell Cycle Analysis

The effects of arnicolide D on the cell cycle were detected by flow cytometry [45]. Briefly, following 24 h or 48 h of treatment with 0–10 µM arnicolide D, cells were harvested. Ice-cold 70% ethanol was added to fix the cells at 4 °C overnight. Cells were then rinsed with ice-cold PBS, and incubated with 25 µL PI and 10 µL RNase for 30 min at 37 °C in the dark. Flow cytometric cell analysis was performed using a BD Accuri C6 flow cytometry system (Becton Dickson Immunocytometry-Systems, San Jose, CA, USA). CellQuest software version 3.3 (Becton Dickson Biosciences, San Jose, CA, USA) was used to determine the proportions of cells at different stages in the cell cycle (G_0_/G_1_, S, and G_2_/M phases). DNA content distribution was analyzed using ModFitLT software v3.2 (ModFit software, Verity Software House, Inc., Topsham, ME, USA).

### 5.5. Annexin V-FITC Apoptosis Assay

The Annexin V-FITC Apoptosis Detection kit was employed for double staining with FITC-Annexin V and PI according to the manufacturer’s introductions [46]. Briefly, cells seeded in 60-mm dishes were treated with 0–50 µM arnicolide D and then collected after 24 h or 48 h. After addition of 195 µL binding buffer, cells were incubated with 5 µL FITC-labelled Annexin V and 10 µL PI at room temperature for 20 min in the dark. The samples were then placed on ice in the dark, and assessed by fluorescence-activated cell sorting (FACS) analysis. Cells were discriminated into viable, necrotic, early apoptotic, and late apoptotic cells.

### 5.6. Morphological Observation

Cells were seeded onto cell culture dishes overnight, and then treated with different concentrations of arnicolide D for 24 h or 48 h. To visualize DNA, the cells were fixed for at least 30 min in 75% ethanol, then stained with 4′,6′-diamidino-2-phenylindole hydrochloride (DAPI) for 10 min at room temperature in the dark, and then rinsed twice with PBS. The stained cells were visualized under a fluorescence microscope (Olympus, Tokyo, Japan) with excitation and emission wavelengths of 350 and 460 nm, respectively.

### 5.7. Western Blot Analysis of the Cell-Cycle-Related Proteins

After treatment with different concentrations of arnicolide D for 24 h or 48 h, cells were harvested for Western blot analysis. Ice-cold RIPA buffer with protease and phosphatase inhibitors was used for cell lysis. Cell lysates were centrifuged at 14,000× *g* for 15 min at 4 °C to pellet cell debris, and supernatants were transferred to a new tube. Protein content of the samples was measured using the Pierce BCA Protein Assay kit (Thermo Scientific, Waltham, MA, USA).

Protein samples were prepared in 5× loading buffer and heated at 98 °C for 5 min. Samples were separated by SDS-PAGE in 8% or 12% polyacrylamide gels using the Bio-Rad Mini-Protean apparatus (Bio-Rad Laboratories, Inc., Hercules, CA, USA) followed by transfer to PVDF membranes by wet transfer. After transfer, membranes were washed with TBST and blocked in 5% skim milk diluted in TBST. Membranes then were washed with TBST three times for 5 min each. After that, membranes were incubated with primary antibody (1:1000) in 5% BSA with gentle agitation overnight at 4 °C. After three washes, membranes were incubated with horseradish peroxidase (HRP)-linked secondary antibody (Cell Signalling Technology, Beverly, MA, USA) (1:2000) in 5% BSA with gentle agitation for 2 h at room temperature, and then washed with TBST three times for 5 min each. Signals were detected with Tanon High-sig ECL Western Blotting Substrate kit, according to manufacturer’s instructions. Finally, signals were quantified using ImageJ (Version 1.52, NIH, Bethesda, MD, USA) and normalized to β-actin.

### 5.8. Statistical Analysis

Statistical analysis of one-way ANOVA was calculated by GraphPad Prism Version 5.0 software (GraphPad Software, Inc.). All data were expressed as means ± standard deviation (SD) of no less than three separate replicates. The criterion for statistical significance was *p* < 0.05.

## Supplementary Materials

The following are available online https://www.mdpi.com/1420-3049/24/10/1908/s1, Figure S1: Chemical structure of arnicolide C, Figure S2: Effects of arnicolide D on proliferation of NPC cells, Figure S3: Effects of arnicolide D on PI3K/AKT pathway (24 h), Table S1: Inhibitory effects of arnicolide C on proliferation of human NPC cells, Table S2: Inhibitory effects of arnicolide D on proliferation of human NPC cells.
