# Supplementary material for: Arnicolide D, from the herb Centipeda minima, Is a Therapeutic Candidate against Nasopharyngeal Carcinoma
Source: Molecules. 2019 May 17;24(10):1908. doi: 10.3390/molecules24101908 (PMC6571971; doi:10.3390/molecules24101908)
Supplement: Supplementary file 1 [file molecules-24-01908-s001.pdf]

molecules

MDPI

Supplementary Materials

Table S1. Inhibitory effects of arnicolide C on proliferation of human NPC cells.

Cell line	IC_{50} (μM)		
	**24 h**	**48 h**	**72 h**
CNE-2	12.3	4.64	3.84
C666-1	8.52	4.87	4.03
CNE-1	11.42	6.81	4.95
SUNE-1	17.69	5.75	3.00
HONE1	15.05	4.42	2.13

Table S2. Inhibitory effects of arnicolide D on proliferation of human NPC cells.

Cell line	IC_{50} (μM)		
	**24 h**	**48 h**	**72 h**
CNE-2	4.26	0.99	0.83
C666-1	2.47	0.91	0.96
CNE-1	3.31	2.19	1.76
SUNE-1	6.22	0.66	0.26
HONE1	9.97	0.19	0.02

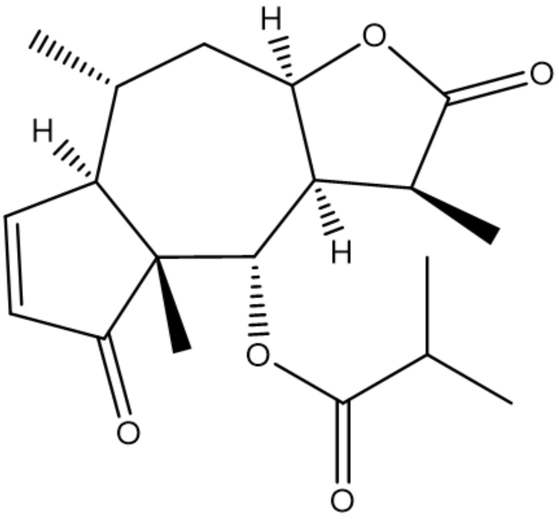

Arnicolide C

Figure S1. Chemical structure of arnicolide C.

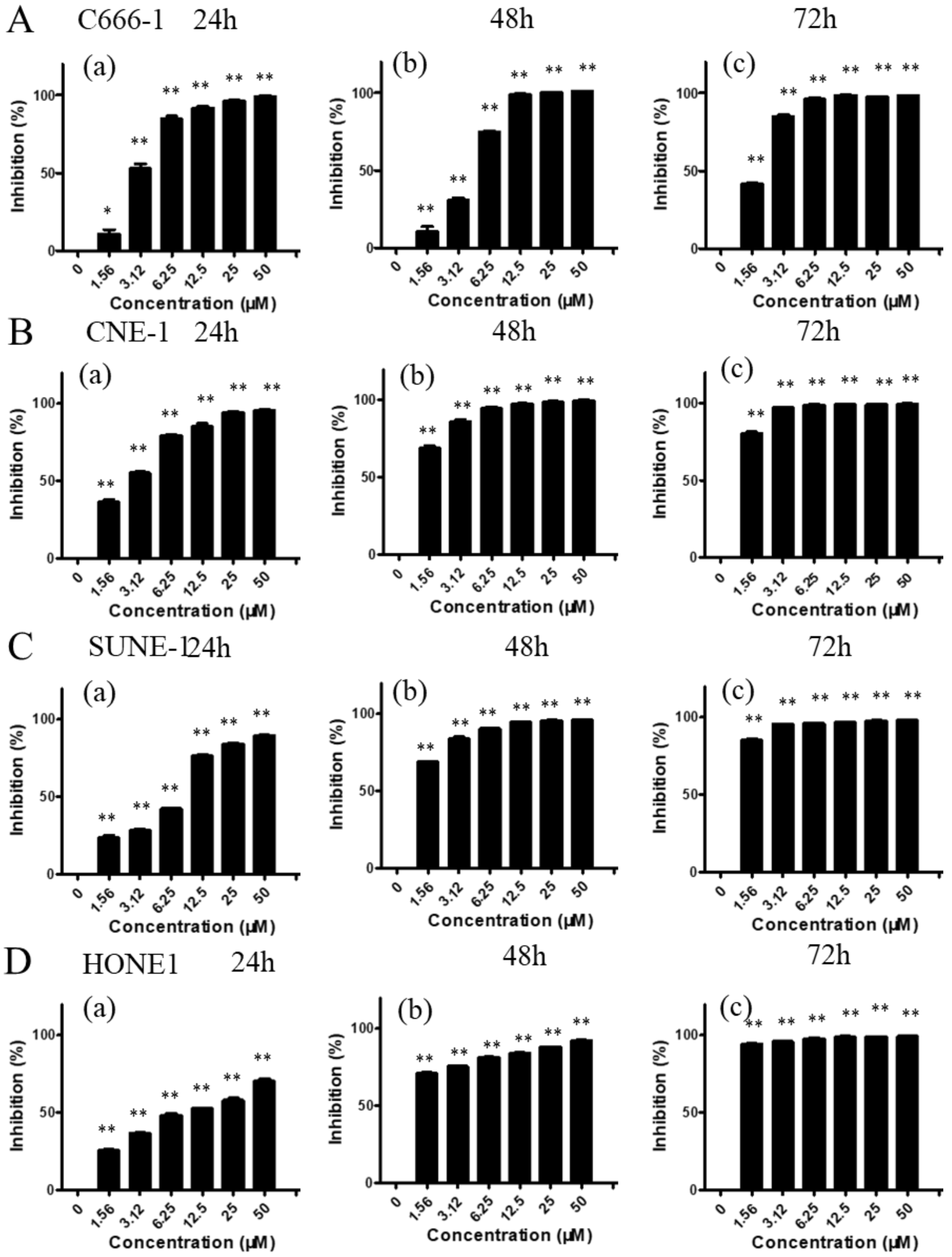

Figure S2. Effects of arnicolide D on proliferation of NPC cells. (A) C666-1, (B) CNE-1, (C) SUNE-1, and (D) HONE1 cells were treated with different concentrations (0-50 μM) of arnicolide D for 24 h, 48 h, or 72 h, after which the MTT assay was used to evaluate their anti-proliferative effects. Cells without drug treatment were used as a control. Data are shown as means ± SD. * $p < 0.05$, ** $p < 0.01$, compared with control.

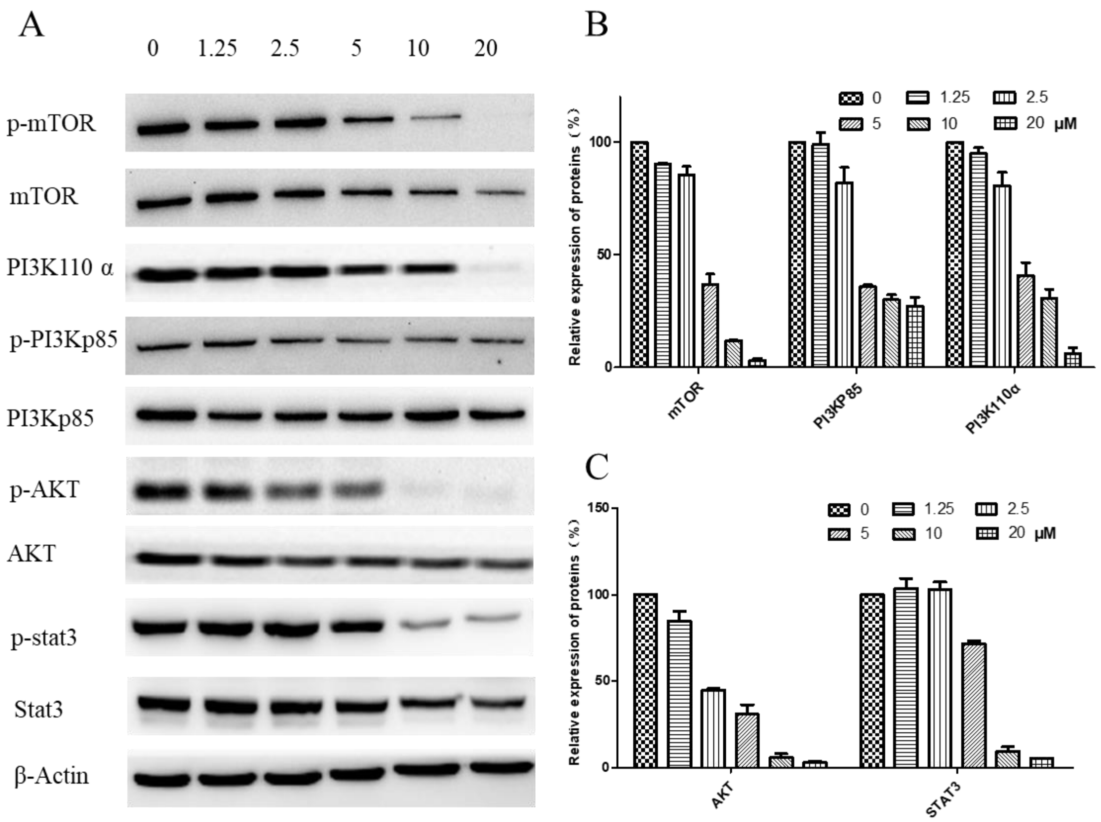

Figure S3. Effects of arnicolide D on PI3K/AKT pathway (24 h). CNE-2 cells were treated with arnicolide D at concentrations of 1.25-20 μM for 24 h. Cell lysates were harvested and subjected to Western blot analysis using antibodies against mTOR and p-mTOR (Ser^{2481}), PI3K p110 α, PI3K p85 and p-PI3K p85 (Tyr^{458}), AKT and p-AKT (Ser^{473}), STAT3 and p-STAT3 (Tyr^{705}). β-actin was used as an internal control. (**A**) Western blot results of 24 h treatments. Bar graphs **(B** and **C)** showing the quantified relative expression of the proteins. Data are expressed as means ± SD. * $p < 0.05$, ** $p < 0.01$ vs. control.
